# Insight into runoff characteristics using hydrological modeling in the data-scarce southern Tibetan Plateau: Past, present, and future

**DOI:** 10.1371/journal.pone.0176813

**Published:** 2017-05-09

**Authors:** Mingyong Cai, Shengtian Yang, Changsen Zhao, Qiuwen Zhou, Lipeng Hou

**Affiliations:** 1 Satellite Environment Center of MEP, Beijing, China; 2 College of Water Sciences, Beijing Normal University, Beijing, China; 3 College of Resource and Environment Sciences, Xinjiang University, Urumqi, China; 4 School of Geography and Environment Science, Guizhou Normal University, Guiyang, China; 5 China-ASEAN Environmental Cooperation Center (China Center for SCO Environmental Cooperation) of MEP, Beijing, China; University of Vigo, SPAIN

## Abstract

Regional hydrological modeling in ungauged regions has attracted growing attention in water resources research. The southern Tibetan Plateau often suffers from data scarcity in watershed hydrological simulation and water resources assessment. This hinders further research characterizing the water cycle and solving international water resource issues in the area. In this study, a multi-spatial data based Distributed Time-Variant Gain Model (MS-DTVGM) is applied to the Yarlung Zangbo River basin, an important international river basin in the southern Tibetan Plateau with limited meteorological data. This model is driven purely by spatial data from multiple sources and is independent of traditional meteorological data. Based on the methods presented in this study, daily snow cover and potential evapotranspiration data in the Yarlung Zangbo River basin in 2050 are obtained. Future (2050) climatic data (precipitation and air temperature) from the Fifth Assessment Report of the Intergovernmental Panel on Climate Change (IPCC-AR5) are used to study the hydrological response to climate change. The result shows that river runoff will increase due to precipitation and air temperature changes by 2050. Few differences are found between daily runoff simulations from different Representative Concentration Pathway (RCP) scenarios (RCP2.6, RCP4.5 and RCP8.5) for 2050. Historical station observations (1960–2000) at Nuxia and model simulations for two periods (2006–2009 and 2050) are combined to study inter-annual and intra-annual runoff distribution and variability. The inter-annual runoff variation is stable and the coefficient of variation (CV) varies from 0.21 to 0.27. In contrast, the intra-annual runoff varies significantly with runoff in summer and autumn accounting for more than 80% of the total amount. Compared to the historical period (1960–2000), the present period (2006–2009) has a slightly uneven intra-annual runoff temporal distribution, and becomes more balanced in the future (2050).

## 1 Introduction

Regional hydrological modeling in ungauged regions has attracted growing attention in water resource management research and has become a grand challenge for theoretical hydrology [1–2]. Rivers in the Himalayan mountain area of the southern Tibetan Plateau often suffer from data scarcity required for hydrological simulation and water resources assessment due to extreme conditions in cross-boundary rivers. This hinders characterizing the water cycle and climate change studies in this area, and finding solutions to international water-resource issues.

Among these rivers, the Yarlung Zangbo River, which originates and flows across the southern Tibetan Plateau, is an important cross-boundary river. Dramatic spatial heterogeneity exists within its drainage area, from the dry, cold upstream area with an elevation > 5000 m, to the humid and hot downstream region ~ 200 m above sea level. Glaciers and permanent snow are features of the high mountainous area in this river basin. The Yarlung Zangbo River and its lower reach, the Brahmaputra in India, flow across four Asian countries including China and India, which have the largest and second largest populations in the world, and the largest and second largest areas in Asia. Water resources from the Yarlung Zangbo River are one of the key factors that influence the relationships between these countries.

Runoff simulations have been carried out in or around the Yarlung Zangbo River basin to determine runoff trends, alterations, and the relationships between hydro-meteorological parameters based on traditional observations [3–6]. Other studies have attempted to evaluate climate change effects in or around the river basin [7,8] using climate models with coarse spatial resolution. A number of studies have also simulated runoff generation in the Himalayan river basin, primarily focusing on the snowmelt process or have used simple regression models based on observations [9–12]. Other water cycle processes, such as evapotranspiration, interflow, or base flow, were not considered in these studies. In the Yarlung Zangbo River, runoff simulations focused on the water cycle and using a physically based distributed hydrological model with high spatial resolution have been rarely completed. Even fewer studies have investigated the runoff characteristics and hydrological response under future climate conditions. There is no analysis of the change in the status of water resources based on distributed hydrological simulation under future climatic situations.

Regional distributed hydrological modeling often suffers from a lack of data due to short historical observations and spatially insufficient observations [13–15]. The parameters and data needed for model validation are often difficult to determine; as a result, the physical basis for models continues to be debated [16]. In the Yarlung Zangbo River basin, traditional observations are insufficient for model simulation due to inadequate meteorological and hydrological station network coverage and the rugged environment over a large area. For this study, multi-source spatial datasets that offer the possibility of overcoming gaps in data availability for distributed hydrological models [17–20] were utilized to prepare model inputs. Numerous studies have focused on deriving key water cycle factors, including precipitation [21–23], evapotranspiration [24,25], land surface temperature [26–28], air temperature [29], snow cover [30,31], and vegetation information [32–34]. Studies combining multi-source spatial data with distributed hydrological models for regional hydrological process simulation have also been carried out [35,36].

In this study, a multi-spatial data based Distributed Time-Variant Gain Model [37] (MS-DTVGM) is applied to the Yarlung Zangbo River basin to simulate runoff. DTVGM [38–41] was proposed on the basis of nonlinear theory [42,43] and the simulation framework was enhanced by taking into account hydrological processes, such as snowmelt, interception, evapotranspiration, surface runoff generation, interflow, and base flow generation. Historical measured average monthly runoff was collected from 1960 to 2000 in the Nuxia section in the Yarlung Zangbo River basin. Using the distributed hydrological model (MS-DTVGM) with physical mechanisms based on multi-source spatial data, runoff processes in the Yarlung Zangbo River basin for the present period (2006–2009) is simulated. Daily potential evaporation and snow cover data in the Yarlung Zangbo River basin in the future (2050) are obtained through the time range extension of MS-DTVGM driven parameters. Utilizing future temperature and precipitation data under the three Representative Concentration Pathway (RCP) situations (RCP2.6, RCP4.5 and RCP8.5) of the fifth Assessment Report of Intergovernmental Panel on Climate Change (IPCC-AR5), this study investigates the hydrological response to climate change. Based on this work, the distribution and variation of the past (1960–2000), present (2006–2009) and future (2050) runoff characteristics are analyzed in the Yarlung Zangbo River basin, southern Tibetan Plateau.

## 2 Materials and methods

### 2.1 Study area

The Yarlung Zangbo River is the highest river in the world with an average elevation of more than 4000 m. It originates from a glacier at an elevation of 5600 m in the southwest Himalayas of the Tibetan Plateau, and is located within the area bounded by 82°00′E-97°07′E and 28°00′N-31°26′N. The river flows through three Asian countries including China, India and Bangladesh, covering an area of 240 000 km^2^.

The Yarlung Zangbo River is 2229 km in length, the longest river in Tibet and the fifth longest river in China. It flows through the dry, flat regions of southwest Tibet with an approximate annual precipitation of just 200 mm before it breaks through the Himalayas near Namcha Barwa peak at 7755 m. In southeast Tibet, it runs through the Great Canyon of Yarlung Zangbo, the deepest valley in the world. The elevation drops abruptly and annual precipitation increases dramatically, reaching up to 6000 mm.

**Fig 1:**
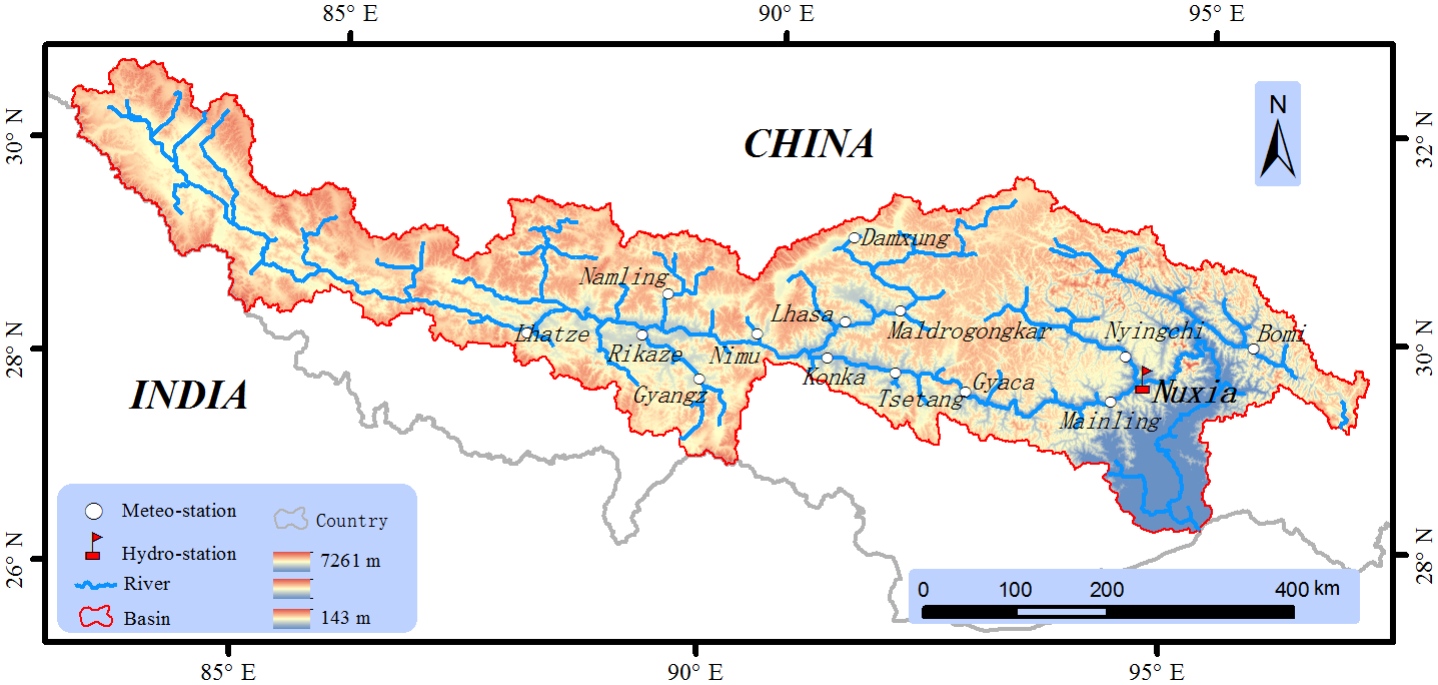
Meteorological and hydrological stations and river network in the Yarlung Zangbo River basin.

Meteorological and hydrological data used for input validation based on spatial data, such as daily precipitation and temperature, were collected from 14 local stations. Daily runoff observations from the Nuxia hydrological station (2006–2009) in the Yarlung Zangbo River basin are used for model calibration and validation. The location of the 14 meteorological stations and Nuxia hydrological station are presented in Fig 1.

### 2.2 MS-DTVGM description and construction

#### 2.2.1 MS-DTVGM description

In this study, a multi-spatial data Distributed Time-Variant Gain Model (MS-DTVGM) is applied to the Yarlung Zangbo River basin for runoff simulation. The MS-DTVGM framework is based on the DTVGM, but some modifications are made to enhance the physical basis of the simulation. In the original DTVGM, the interception by vegetation is calculated using precipitation multiplied by a simple empirical coefficient, which could lead to a bias in water resource assessment over long time scales. In the MS-DTVGM, a well-known interception module [44] is added to assess the interception of vegetation in the rainfall process.

Another important modification concerns the actual evapotranspiration calculation. The Bagrov model [45] used for the calculation in original DTVGM is conceptual and based on the relationship between observed evapotranspiration, potential evapotranspiration, and precipitation, which may not be obtainable in data-scarce regions. In addition, the Bagrov model is designed for calculating multi-year average evapotranspiration rather than for daily simulation. In the MS-DTVGM, the model developed by Kristensen and Jensen [46] is used as a replacement. The Kristensen-Jensen model is part of the global distributed hydrological model MIKE SHE. Moreover, the model is selected because it accounts for vegetation effects in interception and plant transpiration and provides good access to spatial data.

#### 2.2.2 MS-DTVGM construction

Five remote sensing (RS) or Geographic Information System (GIS) spatial datasets are prepared: (1) Moderate Resolution Imaging Spectroradiometer (MODIS) products, including MCD12Q1 (land types), MOD11A1 (land surface temperature), MOD15A2 (leaf area index), MOD13A2 (vegetation index), MOD10A2 (snow cover) and MOD43B3 (Albedo); (2) TRMM rainfall products 3B42 V.6 used as the daily precipitation model (https://disc.sci.gsfc.nasa.gov/); (3) Global Land Data Assimilation System (GLDAS) three-hour surface air temperature dataset; (4) Digital soil map (1:100 000) accompanied by soil properties from the Harmonized World Soil Database (HSWD, http://www.fao.org/); (5) Shuttle Radar Topography Mission (SRTM) digital topographic data (90 m) selected to extract terrain information and the river network. Except for the website where the TRMM products can be obtained was listed above, the left four spatial datasets that can be found according to the description in Multi-Source Spatial Data section [37].

All spatial dataset variables are preprocessed to produce spatially consistent resolution model inputs. The variables are converted to the Albers equal-area projection with a spatial resolution of 1 km and time series data are prepared with a temporal resolution of one day.

In the MS-DTVGM, most parameters are preferentially derived from RS or GIS platform data or from field observations and experiments to avoid an over-parameterized model. In addition, seven important empirical parameters are determined through model calibration. Among them, two parameters are related to surface runoff generation, another two to soil water infiltration of different soil layers, and the remaining three to linear interflow and base flow reservoirs. The soil body is divided into three layers to distinguish runoff (surface runoff, interflow and deep interflow/base flow) generated from different soil parts. The thickness of each soil layer is set to 300 mm, 400 mm and 400 mm from top to bottom respectively.

Three criteria are selected for evaluating simulated model runoff performance. The definition of each is given below (Eqs 1, 2 and 3).

1. Water balance index ROE:
  $$\text{ROE}=\frac{\sum_{t=1}^{T}Q_{o}^{t}}{\sum_{t=1}^{T}Q_{m}^{t}}$$ (1)
2. Coefficient of efficiency NSE [47]:
  $$\text{NSE}=1-\frac{\sum_{t=1}^{T}{\left(Q_{o}^{t}-Q_{m}^{t}\right)}^{2}}{\sum_{t=1}^{T}{\left(Q_{o}^{t}-{\bar{Q}}_{0}\right)}^{2}}$$ (2)
3. Root mean square error (RMSE):
  $$\text{RMSE}=\sqrt{\frac{\sum_{t=1}^{T}{\left(Q_{o}^{t}-Q_{m}^{t}\right)}^{2}}{n-1}}$$ (3)

Where *Q*_*o*_ is observed runoff (m^3^/s), *Q*_*m*_ is simulated runoff (m^3^/s), $\bar{Q_{o}}$ is the mean of the observed value (m^3^/s), t stands for each comparative time, and T is the length of the data time series. The RMSE and R^2^ indexes can also be used for evaluating the accuracy of other parameters.

### 2.3 Multi-source spatial data-based inputs

#### 2.3.1 Precipitation

Precipitation is one of the most important inputs in runoff simulation because it controls runoff generation and evapotranspiration. A growing number of methods based on remote sensing data have emerged for estimating precipitation, and most utilize thermal infrared (TIR), microwave, and radar sensors alone or in combination. In addition, a variety of precipitation products based on satellite observations, such as Tropical Rainfall Measuring Mission (TRMM), Pathfinder (jointly initiated by NOAA and NASA in 1990 through the “Early-EOS Pathfinder Data Set Activity”), and the Global Precipitation Climatology Project (GPCP) are available to meet various data requirements. For this study, TRMM precipitation products are selected and their accuracy in the Yarlung Zangbo River basin is validated using observations from 14 meteorological stations. The results are shown in Table 1.

**Table 1:** Validation of TRMM precipitation at daily and monthly time scales.

Station	D-*R*^*2*^	ROE	M-*R*^*2*^	Station	D-*R*^*2*^	ROE	M-*R*^*2*^
Damxung	0.18	0.91	0.88	Maldrogongkar	0.25	0.92	0.88
Lhatze	0.22	1.00	0.88	Tsetang	0.23	1.09	0.83
Namling	0.23	0.79	0.86	Gyangz	0.17	1.61	0.8
Rikaze	0.25	0.9	0.94	Bomi	0.09	0.96	0.5
Nimu	0.25	1.00	0.84	Gyaca	0.25	1.01	0.84
Konka	0.25	0.94	0.84	Nyingchi	0.20	1.12	0.89
Lhasa	0.24	1.02	0.91	Mainling	0.17	1.08	0.84

Note: D-*R*^*2*^ and M-*R*^*2*^ indicate the *R*^*2*^ value of the daily and monthly test, respectively.

Table 1 shows that the R^2^ values at the 14 stations vary from 0.09 to 0.25 at daily validation and the R^2^ value at more than 70% of stations are around 0.23. The poorest value is at Bomi station, while the highest is at Rikaze and four other stations. Similarly, the monthly validation at most stations show good performances, with an R^2^ of more than 0.8, the weakest and strongest R^2^ are again at Bomi and Rikaze with values of 0.5 and 0.94, respectively. The ROE index, which indicates the difference between TRMM precipitation and station observations, shows a satisfactory performance. The ROE indices at 11 of the 14 stations show no more than 10% deviation from observations. The TRMM precipitation at Nyingchi is 12% above observations and just two stations, Namling and Gyangz, have deviations of more than 15%.

#### 2.3.2 Air temperature

Surface air temperature is another key parameter because it has a significant influence on both evapotranspiration and snowmelt. In ungauged regions, such as the study region, few station measurements can be used to interpolate the distribution of atmospheric temperature, especially under complicated terrain conditions.

The GLDAS surface air temperature data are used to obtain the daily average air temperature with a spatial resolution of 1 km. GLDAS datasets provide near-land surface air temperature data with a high temporal resolution of three hours and a coarse spatial resolution of 0.25 degrees. The average daily value is calculated as the mean of eight three-hour instant values. The GLDAS temperature data are downscaled to a spatial resolution of 1 km using a simple and logical method (described below) before they are used as model inputs [48].

The downscaling process relies on two assumptions. First, surface air temperature is primarily influenced by elevation, and so other factors can be ignored during downscaling. Second, the GLDAS surface air temperature value for each grid is equal to the temperature at the average elevation of the grid. We introduce a temperature-reduction rate, fixed at 0.65 degrees per 100 m to quantify the extent of temperature change with elevation (Eq 4). Along with the prerequisites described above, compared to GLDAS, the core of this method is the introduction of a higher spatial resolution (1 km) Digital Elevation Model (DEM) to refine the spatial distribution of the GLDAS temperature value.

ΔT=ΔH×δ (4)

Where Δ*T* stands for the temperature difference between location A and B, Δ*H* indicates the altitude difference between A and B, and δ is the temperature-reduction rate.

The downscaling results are validated using the observed daily temperatures from 2006 to 2009 at all 14 stations. The validation results show that the downscaled GLDAS temperature data correlate well with station measurements; the accuracies of the downscaled temperature data at each station are provided in Table 2.

**Table 2:** Validation of downscaled GLDAS daily average temperatures at 14 stations.

Name	Elevation (m)	RMSE (°C)	*R*^*2*^
Damxung	4200	2.37	0.92
Lhatze	4000	4.81	0.88
Namling	4000	2.75	0.91
Rikaze	3836	2.62	0.87
Nimu	3809	2.31	0.9
Konka	3555	2.56	0.88
Lhasa	3648	2.34	0.9
Maldrogongkar	3804	2.52	0.9
Tsetang	3551	2.43	0.91
Gyangz	4040	2.39	0.87
Bomi	2736	2.78	0.91
Gyaca	4260	2.57	0.88
Nyingchi	2991	2.67	0.87
Mainling	2950	2.22	0.89

Table 2 shows the RMSEs for 13 of the 14 stations range from 2.22°C to 2.78°C and the R^2^ ranged from 0.87 to 0.91. Lhatze alone has an RMSE of 4.81°C, which is much higher than that of the other stations. Though downscaling is based on elevation and an assumed relationship between elevation and air temperature, no obvious systematic distribution of the RMSE following the elevation distribution is found. Lhatze and Namling have nearly identical elevations, but the RMSEs at these two stations differ significantly. The RMSE values at Bomi and Namling, and at Gyaca and Konka, are much closer considering that for each pair, their elevation differs by approximately 1300 m.

#### 2.3.3 Potential evapotranspiration

The potential evapotranspiration derived from spatial data is based on the Priestley-Taylor equation [49] (Eq 5), in which the net radiation Rn is the dominant parameter. Because Rn can be obtained from satellite observations using existing methods, this method is more suitable for ungauged regions.

$$ET_{p}=\alpha \left(\frac{R_{n}-G}{\lambda }\right)\left(\frac{\Delta }{\Delta +\gamma }\right)$$ (5)

Where *ET*_*p*_ ETp is potential evapotranspiration (mm day^-1^); *R*_*n*_ and *G* are net radiation and ground heat flux (MJ m^-2^ day^-1^), respectively; Δ is the slope vapor pressure curve (kPa °C^-1^) and is determined by air temperature; *γ* is the psychrometer constant (kPa °C^-1^), which is determined by air pressure; *λ* is the latent heat of evaporation (2.45 MJ kg^-1^), which is used to transfer evaporation from an energy unit to a water quantity unit; *α* is set to a value of 1.26 as advised by Priestley and Taylor [49].

Rn is the difference between the input and output of ground surface radiative energy, controlling water transmission and exchange. We calculate Rn using MODIS products and an energy balance equation (Eq 6):

$$ING=R_{s}↓-R_{s}↑+R_{L}↓-R_{L}↑=\left(1-\alpha \right)R_{s}↓+R_{L}↓-R_{L}↑$$ (6)

Where *α* is albedo; *R*_*s*_↓ is the down-welling surface short-wave radiation flux (w m^-2^); *R*_*L*_↓ and *R*_*L*_↑ are the down-welling and up-welling long-wave radiation flux (w m^-2^), respectively. The technology described in Su [50] is used to obtain the distribution of G in the study area.

The instantaneous potential evapotranspiration can be derived using the methods mentioned above. Because the average daily ETp is needed as an input for MS-DTVGM, a temporal scale transformation is necessary. In this study, the method described by Xie [51] is used to obtain daily potential evapotranspiration, with the assumption that the method used for farmland is appropriate for other types of land cover.

Daily potential evapotranspiration estimates are not validated due to limited direct observation. Instead, the validity of the spatial data-based potential evapotranspiration is evaluated implicitly through runoff predictions produced by the hydrological model.

#### 2.3.4 Vegetation parameters

Three important vegetation parameters are used in the water cycle simulation: leaf area index (LAI), vegetation cover, and root depth. LAI is obtained from the MODIS product and the remaining two parameters are derived on the basis of LAI.

Vegetation cover, which provides information on the growth and health of vegetation, is determined using the ratio between the vertically projected area of the vegetation above the land surface and grid area. Satellite observation and other remote sensing measurement methods provide a mechanism for retrieving large-scale vegetation cover data that cannot be obtained using conventional sampling methods. In this study, LAI is introduced to calculate vegetation cover using a relationship between vegetation cover and LAI (Eqs 7 and 8) proposed by Nilson [52].

$$VF=1-e^{-k\times LAI}$$ (7)

k=Ω·K (8)

Where *VF* is vegetation cover; *k* is the coefficient concerned with vegetation geometric structure; Ω is the aggregation index of each vegetation type; *K* can be calculated using Eq 9, where z is the solar zenith angle.

K=0.5/cosz (9)

Another important vegetation index is the root depth related to vegetation transpiration. The traditional method for obtaining root depth is through field sampling, but this method is not practical on a regional basis. In this study, a method described by Andersen [53] is used to simulate annual root-depth variation with the assumption that yearly root depth of multi-year trees can be fixed. In contrast, the root depth of annual herbs and crops varies with LAI (Eq 10).

$$Rd_{i}=Rd_{\text{max}}\frac{LAI_{i}}{LAI_{\text{max}}}$$ (10)

Where *Rd*_*i*_ is simulated root depth; *Rd*_max_ is the maximum annual root depth, which depends on vegetation type; *LAI*_*i*_ is LAI at the simulated time; *LAI*_max_ is the maximum LAI value in a year, which also varies with vegetation type.

#### 2.3.5 Future model input preparation

MS-DTVGM, a distributed hydrological model based on physical mechanisms, needs many parameters and inputs to simulate the rainfall-runoff process. In order to study hydrological processes under future climate conditions in the Yarlung Zangbo River basin, future precipitation and air temperature data provided by the IPCC-AR5 are necessary, as well as future potential evaporation, vegetation and soil information.

1. Future air temperature and precipitation
  Climate forecast data in 2050 are selected from IPCC-AR5 under three climate conditions (RCP2.6, RCP4.5 and RCP8.5) as inputs for the MS-DTVGM simulation in the Yarlung Zangbo River basin. The temperature and precipitation data for 2050 are provided directly by the IPCC-AR5 interpolated at 1 km spatial resolution and 1 day temporal resolution using the same downscaling method previously described.
2. Future potential evaporation
  Future potential evaporation is determined by applying the statistical relationship between present potential evaporation and primary factors, temperature, precipitation and elevation, to potential evaporation and future primary factors. Utilizing multiple stepwise regressions, the relationship between potential evaporation (ETp) is simulated for the present period and primary factors. After stepwise regression analysis, the final statistical model (Eq 11, R^2^ = 0.82 and RMSE = 0.63) contains only the average daily temperature (T_air_, °C) as the predictor.
  $$ET_{p}=1/\left(0.02\times Tair+7.94/Tair-0.55\right)+1.97$$ (11)
3. Future vegetation and soil information
  Considering the low development and weak human activity effect in the Yarlung Zangbo River basin, little change in vegetation and soil information is assumed between the present and 2050. Therefore, the same vegetation and soil information data are used for future simulations as for the present period.

## 3 Past runoff characteristics in the southern Tibetan Plateau

### 3.1 Intra-annual runoff variation and analysis

**Fig 2:**
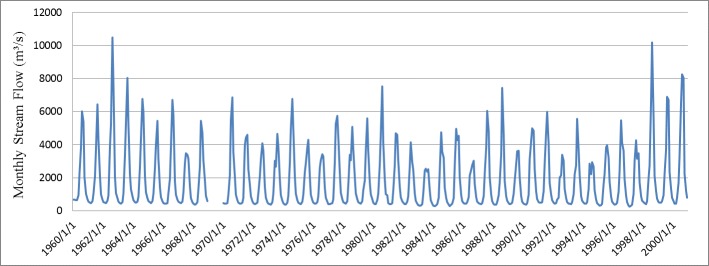
Monthly stream flow observations at Nuxia hydrological station from 1960 to 2000.

The intra-annual runoff is significantly variable in the Yarlung Zangbo River basin, as shown in Fig 2. Based on the statistics, the lowest average monthly stream flow is 423 m^3^/s in February, and the highest average monthly stream flow is 13 times this value at 5335 m^3^/s in August. The largest fluctuation in one year appears in 1998, with the highest monthly stream flow (10,183 m^3^/s in August) being 24.5 times this value (416 m^3^/s in March). According to monthly stream flow data analysis for 40 years (1960–2000), the Coefficient of Variation (CV) of average monthly stream flow is 0.98, which is approximately five times the CV of inter-annual stream flow. This variation shows the extreme distribution in the Yarlung Zangbo River basin.

**Fig 3:**
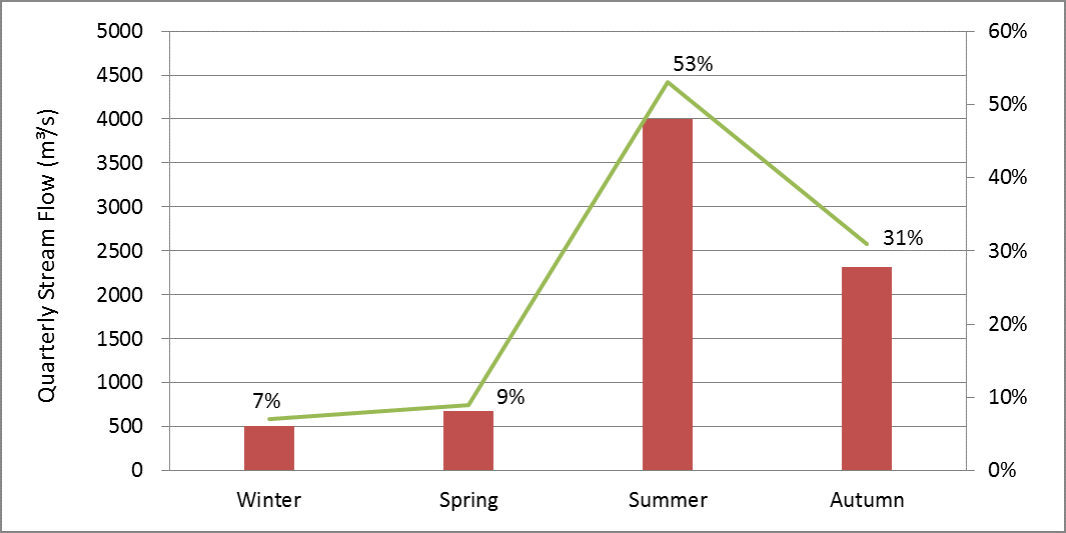
Quarterly stream flow observations at Nuxia hydrological station from 1960 to 2000.

Historical quarterly stream flow distribution at Nuxia is shown in Fig 3. Most runoff in the Yarlung Zangbo River basin is in the summer and autumn, the sum of the two accounts for 84% of annual runoff. The runoff in summer accounts for 53% of annual runoff. There are three reasons for this result. First, runoff in the Yarlung Zangbo River basin is mainly derived from precipitation and snowmelt. Warm moist air flows north from the Indian Ocean, bringing a large amount of precipitation and rapidly increases river runoff. Second, the mountain snow melting process is relatively strong during summer and autumn. The effect of snowmelt also plays an important role in increasing river runoff. Third, river runoff decreases because of shrinking precipitation and temperature during winter and spring. However, a small runoff peak can be formed in spring due to temporary winter snow melting rapidly as temperature rises.

### 3.2 Inter-annual runoff variation and analysis

**Fig 4:**
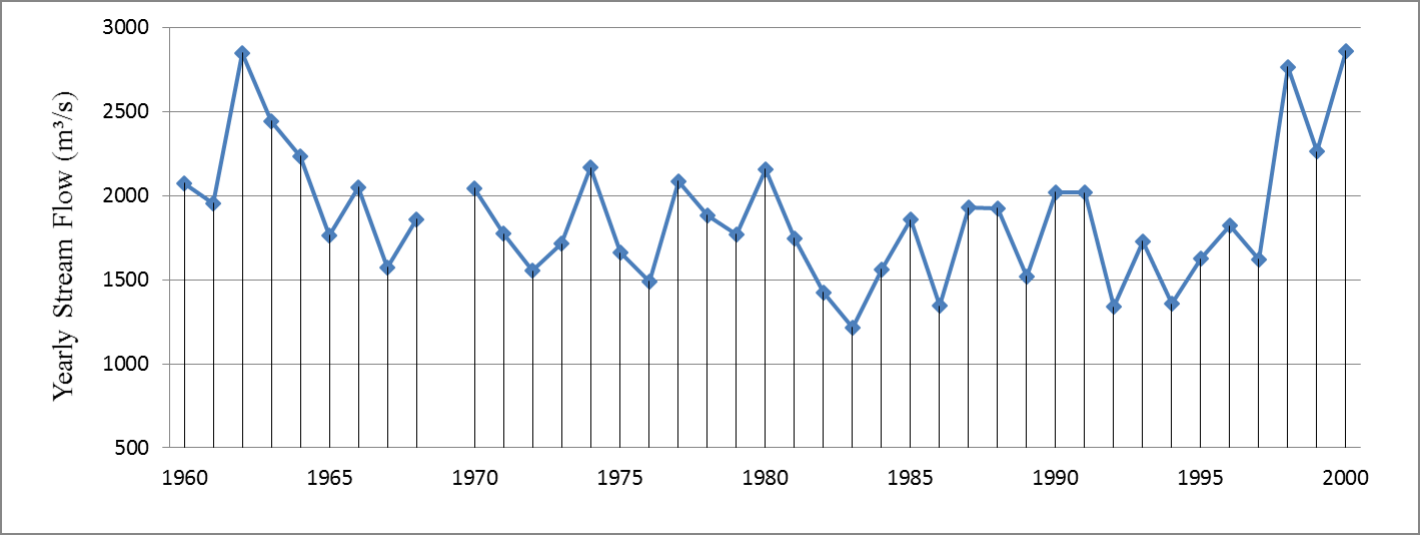
Yearly stream flow observations at Nuxia hydrological station from 1960 to 2000.

Two obvious fluctuations in runoff in the 40 years from 1960 to 2000 are shown in Fig 4. The first fluctuation is characterized by a descent from 2850 m^3^/s to 1573 m^3^/s in 1962–1967, declining by 45%. The second is characterized by a steep climb from 1624 m^3^/s to 2862 m^3^/s between 1995 and 2000, rising by 76%. Average annual runoff in the Yarlung Zangbo River basin is relatively stable from 1967 to 1995. On the whole, the annual runoff at Nuxia hydrological station is relatively flat from 1960 to 2000. The average annual stream flow is 2862 m^3^/s, and the lowest value is only 43% of the 1217 m^3^/s peak in 1983. According to the calculation, average annual runoff has a CV of 0.21 for 1960–2000, showing a stable inter-annual variability in the Yarlung Zangbo River basin.

There are three reasons for the stable inter-annual runoff variability. First, observation data derived from the Nuxia hydrological station, located in the lower reaches of Yarlung Zangbo River basin, represent the whole condition of the basin. In addition, the tributary catchment area is so small that a sharp fluctuation can be weakened in the process of transmission. Therefore partial changes cannot be reflected in measurements at the monitoring section for the basin. Second, the Yarlung Zangbo River basin has a vast area and relatively stable climate. An extremely abnormal climate in a large area of the southern Tibetan Plateau rarely occurs. A partially abnormal climate is hidden by the entire hydrological process of the Yarlung Zangbo River basin. Third, water resources are rich because of plentiful precipitation in the river basin. Normal climatic fluctuations have little effect on runoff, which is different from rivers in arid areas.

## 4 Present runoff simulation and analysis in the southern Tibetan Plateau

### 4.1 Present runoff simulation

The daily discharge data from 2006 to 2009 at the Nuxia hydrological station are used for modeling calibration using the one-site calibration method [53] and modeling validation. Observations from 2006 to 2007 are used for parameter calibration while observations from 2008 to 2009 are used for modeling runoff validation. In addition, the model inputs based entirely on multi-source spatial data are verified to reduce the uncertainty from data integration and parameter inversion.

Modeling simulations for the study are carried out for June, 2005 to December, 2009. The statistical analysis does not begin until 2006, because no valid data were collected prior to 2006. Although simulations for 2005 are not included in analysis, they are carried out to eliminate the influence of certain parameters on initial values and stabilize the model simulation. In the calibration, the Nash-Sutcliffe Efficiency (NSE) is used as the main objective function. Details of the seven calibration variables in this study and their optimized values are listed in Table 3.

**Table 3:** Parameters and their optimized values for MS-DTVGM in the Yarlung Zangbo River.

Parameters	Lower bound	Upper bound	Model value	Descriptions
g1	0	1	0.6	Surface runoff generation
g2	1	10	6	
Kr	0	1	0.02	Top soil layer flow efficiency (%)
Km	0	1	0.03	Middle soil layer flow efficiency (%)
Kg	0	1	0.01	Deep soil layer flow efficiency (%)
fc1	0	1	0.3	Top soil layer infiltration rate (%)
fc2	0	1	0.2	Middle soil layer infiltration rate (%)

**Fig 5:**
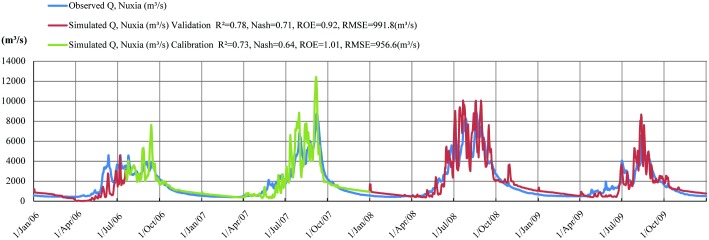
The observed and simulated runoff hydrographs at Nuxia for both calibration and validation periods.

A fairly good consistency between simulated daily runoff and observations is shown in Fig 5, particularly in consideration of the purely spatial data. Based on time series simulations, four accuracy assessment criteria are calculated for both the calibration and validation periods. During the calibration period, R^2^ is 0.73, which demonstrates a good correlation between observed and simulated daily stream flow. The ROE index is 1.01, indicating that the average observed stream flow almost matches the average simulated stream flow with a deviation of just 1%. The RMSE is 956.6 m^3^/s and the Nash-Sutcliffe Efficiency (NSE) is 0.64, meaning that the simulated daily runoff curve effectively matches the observed curve. These criteria are established so that stream flow at the Yarlung Zangbo River basin is accurately simulated using the MS-DTVGM on the basis of multi-spatial data. These four criteria also perform satisfactorily for the validation period. The R^2^ and NSE for the validation period are 0.78 and 0.71, which show a better performance than the calibration period. The RMSE is 991.8 m^3^/s, which is nearly identical to the RMSE in the calibration period. The ROE index is 0.92, which implies more deviation during the validation period.

Fig 5 indicates that, although the variation in the simulated runoff curve is consistent with observations, the simulated curve (in green and red) fluctuates more severely than the observed curve (in blue), with numerous data extreme values. All inputs for the simulated runoff are from multi-source spatial datasets with a range in spatial and temporal resolutions, and often with significant missing data. The exclusive use of straightforward methods to address the time scale transformation and interpolation contribute to the extreme values in the simulation. The mismatch between daily TRMM precipitation and observations also accounts for the difference between two daily runoff curves. Notable underestimates are found in the simulation during May and June each year for both the calibration and validation periods. The first peak in the observed runoff curve that occurs in May and June is due to a sharp rise in snowmelt. The underestimation in daily runoff for this period may have resulted from a less accurate snowmelt calculation. The TRMM precipitation is validated using observations and no clear deviations are found for this time during the simulation period. Further work is needed to verify this assumption.

The overall accuracy of daily runoff simulation is also calculated. The R^2^ for the entire simulation period from 2006 to 2009 is 0.76 and the NSE is 0.68; both show satisfactory accuracy. The overall ROE index is 0.96, meaning that the modeled runoff overestimates observations by 4% for the simulation period.

### 4.2 Present runoff characteristics analysis

Yearly stream flow simulations at Nuxia hydrological station from 2006 to 2009 are shown in Table 4. The CV of the inter-annual runoff is 0.27 from 2006 to 2009, which is higher than 0.21 from 1960 to 2000. The lowest stream flow is 1348 m^3^/s in 2006 and the highest is 2466 m^3^/s in 2008.

**Table 4:** Simulated monthly stream flow at Nuxia hydrological station from 2006 to 2009 (m^3^/s).

Year	Winter			Spring			Summer			Autumn			Annual
	Dec	Jan	Feb	Mar	Apr	May	Jun	Jul	Aug	Sep	Oct	Nov	
2006	849	841	654	369	58	397	1307	2379	3135	3483	1642	1063	1348
2007	1039	687	547	430	622	578	1292	4387	5108	5197	1599	1237	1893
2008	1181	898	702	559	473	855	2957	6847	6866	4394	2181	1688	2466
2009	846	977	802	645	616	771	1248	2474	5004	2293	1579	1061	1526
Season	835			531			3583			2284			

As with the runoff variation in the historical period, there is high variability in the present period, as shown in Table 4. The CV of monthly average stream flow is 0.95 simulated using MS-DTVGM, showing a tremendous variation in the Yarlung Zangbo River basin in the present period (2006–2009).

Quarterly stream flow simulations at Nuxia hydrological station from 2006 to 2009 are shown in Table 4. Most runoff is in summer and autumn, the sum of the two accounts for 82% of annual runoff. The runoff in summer accounts for 50% of annual runoff, slightly less than the 53% in the historical period. The sum of winter and spring runoff accounts for 19% of annual runoff, which is higher than the 16% in the historical period. This phenomenon is more obvious in winter; the runoff rises from 7% in the historical period to 12% in the present period. Spring runoff falls slightly, accounting for 7% of annual runoff. Autumn runoff increases slightly, accounting for 32% of annual runoff.

## 5 Future runoff simulation and analysis in the southern Tibetan Plateau

### 5.1 Future runoff simulation

**Fig 6:**
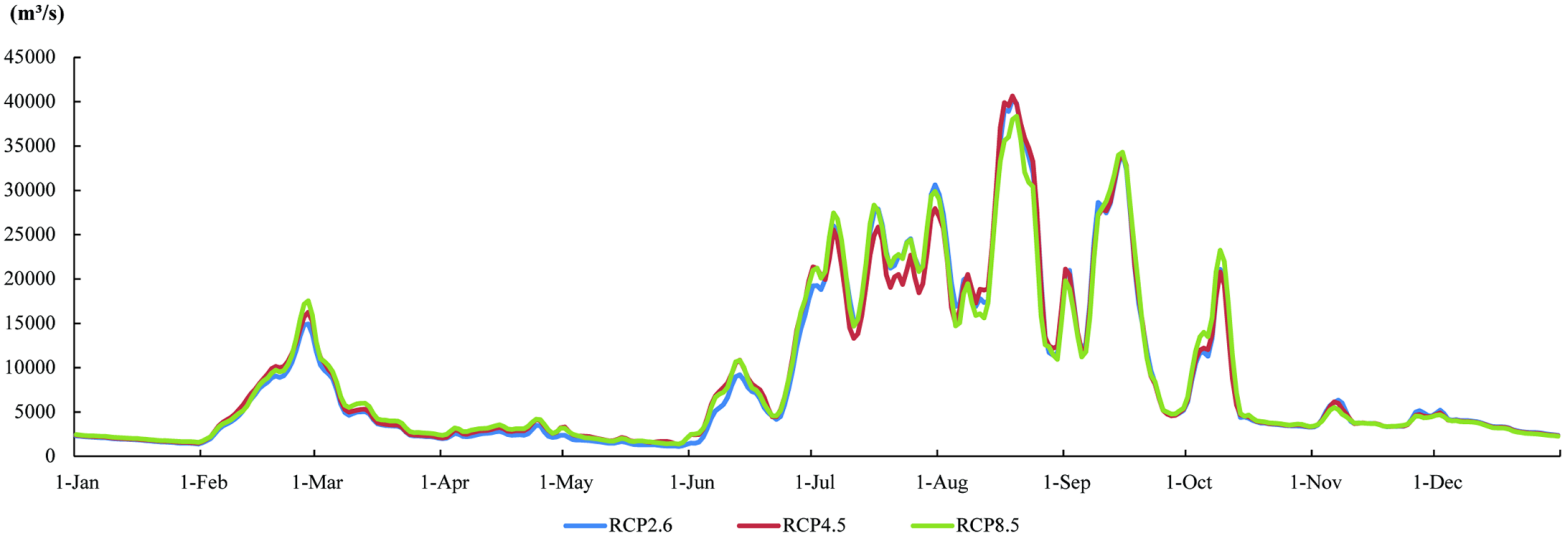
Daily runoff simulation at Nuxia hydrological station under three RCP scenarios in 2050.

The daily scale runoff process curve for 2050 in the Yarlung Zangbo River, simulated using MS-DTVGM, is shown in Fig 6. The figure shows that runoff obviously increases at Nuxia hydrological station in 2050 based on IPCC-AR5 data under three climatic conditions (RCP2.6, RCP4.5 and RCP8.5). The overall runoff simulation values in 2050 are greater than the historical period (1960–2000) and remote sensing monitoring period (2006–2009). The average annual runoff is about 8846 m^3^/s, which is 4.7 times the historical average annual runoff (1876 m^3^/s). The distribution of intra-annual runoff in 2050 has the same pattern as the historical and remote sensing monitoring periods. The proportion of winter and spring runoff in annual runoff is small, while summer and autumn runoff accounts for a larger proportion. The runoff appears as an obvious peak value between February and March in 2050. Compared to the historical and remote sensing monitoring periods, the volatility of runoff in this period clearly increases. This indicates that the spring flood is more prevalent under future climate conditions. The peak runoff value appears in August, 40654 m^3^/s, and the valley runoff value in May is 1385 m^3^/s.

### 5.2 Future runoff characteristics analysis

A preliminary analysis of the cause of significant runoff increase at Nuxia hydrological station in 2050 has been studied.

Compared with historical observations and present simulations, runoff also changes in 2050 in the Yarlung Zangbo River basin (Table 5). However, the runoff distribution is different, with the lowest value appearing in January or May and the highest value appearing in August. Compared to monthly stream flows in the historical and present remote sensing periods, both the lowest and highest values are significantly elevated under future climate conditions. The CVs of monthly average runoff under three RCP scenarios are between 0.87 and 0.93, which are close to 1 but are smaller compared to the historical and present remote sensing periods. This indicates that inter-monthly variation is mitigated in 2050 in the Yarlung Zangbo River basin.

**Table 5:** Monthly stream flow simulations using MS-DTVGM under three RCP scenarios in 2050.

Year	Month	RCP2.6 (m^3^/s)	RCP4.5 (m^3^/s)	RCP8.5 (m^3^/s)
2050	January	1835.0	1920.1	1993.8
	February	7295.1	8018.5	7945.7
	March	4727.0	4998.1	5396.7
	April	2513.8	2958.6	3167.5
	May	1509.4	1938.2	1877.9
	June	6926.5	8135.5	7979.4
	July	22555.3	20927.7	22967.3
	August	24458.4	24586.2	22886.3
	September	17608.3	17524.9	17560.0
	October	7399.5	7473.8	8281.0
	November	4248.7	4158.0	4047.4
	December	3442.8	3353.9	3295.1
Coefficient of Variation (CV)		0.93	0.88	0.87

**Fig 7:**
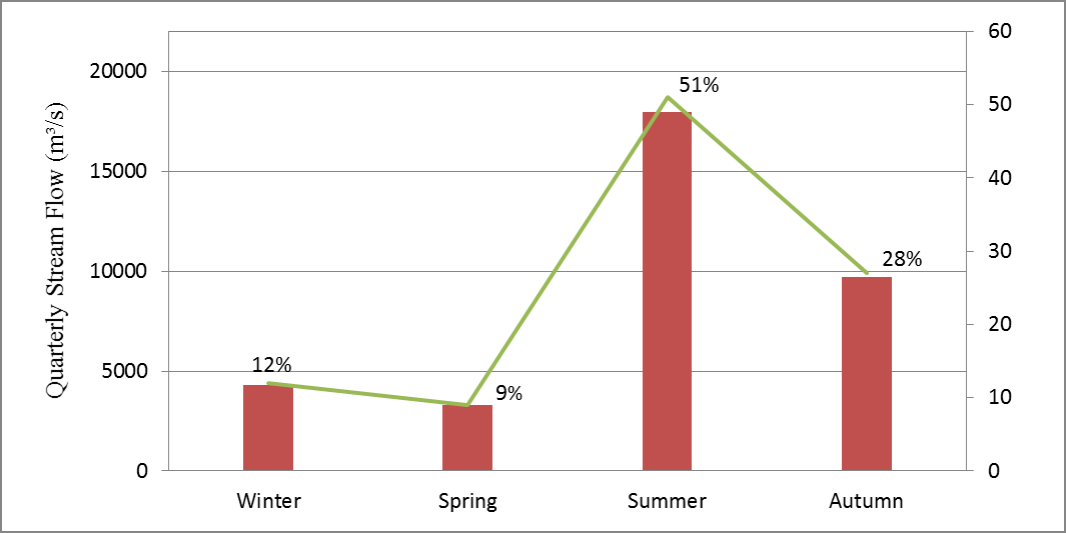
Quarterly stream flow distribution at Nuxia in 2050 (the average of 3 RCP types).

According to Fig 7, most runoff exists in the summer and autumn, the sum of the two accounts for 79% of annual runoff. The runoff in summer accounts for 51% of annual runoff, which is smaller than the 53% found in the historical period but is higher than the 48% found in the present remote sensing monitoring period. The sum of winter and spring runoff accounts for 21% of annual runoff, which higher compared to 16% found in the historical period and 18% in the present period. This phenomenon is more obvious in winter; runoff rises from 7% in the historical period and 10% in the present period to 12% in the future period. Spring runoff maintains 9% of annual runoff. Autumn runoff falls significantly from 31% in the historical period, and 33% in the present period, to 28% in 2050. The runoff distribution in the basin for the four seasons becomes more balanced.

## 6 Conclusions

This study collects historical measured data of average monthly runoff from 1960 to 2000 in the Nuxia section. Using a time range extension of MS-DTVGM driven parameters, the study provides daily potential evaporation and snow cover data for the Yarlung Zangbo River basin in the future (2050). Using the distributed hydrological model (MS-DTVGM) and physical mechanisms based on multi-source spatial data, the runoff process in the Yarlung Zangbo River basin in the present period (2006–2009) is simulated. Utilizing future temperature and precipitation data under three IPCC-AR5 scenarios (RCP2.6, RCP4.5 and RCP8.5), this study investigates the hydrological response to climate change. Based on this work, the distribution and variation of past (1960–2000), present (2006–2009) and future (2050) runoff characteristics are analyzed in the Yarlung Zangbo River basin in the southern Tibetan Plateau. The four primary conclusions of this study are as follows.

1. Inter-annual runoff fluctuation is weak and intra-annual runoff changes dramatically in the Yarlung Zangbo River basin. The range in inter-annual runoff fluctuation range for three periods (1960–2000, 2006–2009, 2050) is small; the CV is between 0.21 and 0.27. However, intra-annual runoff changes dramatically, the CV is between 0.87 and 0.98. The intra-annual runoff varies significantly, and runoff in summer and autumn accounts for more than 80% of the total.
2. Runoff variations in the Yarlung Zangbo River basin are small in the three IPCC-AR5 climate scenarios (RCP2.6, RCP4.5 and RCP8.5). According to the study results, daily runoff variance caused changing climate conditions in the three RCP models is small. This indicates that the Yarlung Zangbo River basin is not sensitive to differing climate scenarios in 2050.
3. Compared to the historical and present periods, runoff will clearly increase under future climate conditions. This study shows that the average annual runoff and peak flow in 2050 are higher than those in the present condition. There are three causes. First, both peak and average precipitation values in 2050 provided by IPCC-AR5 are much higher than the model driven precipitation data for the present period. Second, the average value within a year and average value within the snowmelt period are both much higher than values from the model driven data in the present period. Third, the snow-covered area calculated using the algorithm is close to the current snow-covered area. The combined effort of these three factors eventually leads to a simulation runoff value that is far higher than those in the historical and present period.
4. The Yarlung Zangbo River basin becomes moister and the seasonal distribution runoff in the basin becomes more uniform. The runoff data simulated by three different kinds of climatic condition models show that the winter in the Yarlung Zangbo River basin becomes moister and runoff increases. The proportion of summer and autumn runoff declines for the whole year in the future. Compared to the historical period (1960–2000), intra-annual runoff temporal distribution is slightly uneven in the present period (2006–2009), and becomes more balanced in the future period (2050).
